# Circulating Progenitor Cells in Regenerative Technologies: A Realistic Strategy in Bone Regeneration?

**DOI:** 10.23937/2469-570x/1410026

**Published:** 2016-02-26

**Authors:** Jessica B. Chang, Justine C. Lee

**Affiliations:** Division of Plastic and Reconstructive Surgery, UCLA David Geffen School of Medicine, Los Angeles, CA

**Keywords:** Cellular progenitors, Mesenchymal Stem Cells (MSCs), Osteogenesis

## Abstract

Strategies in skeletal regeneration research have been primarily focused on optimization of three components: cellular progenitors, biomaterials, and growth factors. With the increased understanding that circulating progenitor cells exist in peripheral blood, the question arises whether such cell types would allow for adequate osteogenesis and mineralization. In this review, we discuss the current literature on circulating progenitor cells in *in vitro* and *in vivo* studies on bone regeneration.

## Short Review

The regeneration of bone is a complex physiological process involved in fracture healing as well as defects created by trauma, infection, tumor resection, congenital abnormalities, and impaired or insufficient regeneration [1]. Various bone regeneration and repair strategies exist to augment surgical reconstructive procedures, including use of alloplastic and allogenic materials, distraction osteogenesis, osteoconductive scaffolds, and bone morphogenetic proteins. Despite the numerous options, the gold standard has remained autologous bone grafting [1,2]. However, limitations to this approach, particularly donor site morbidity and an inadequate supply of graft material, have led researchers to turn to cell-based tissue engineering strategies as a novel and attractive alternative [3].

Osteoprogenitor cells have been derived from sources such as bone marrow (BM) mesenchymal stem cells (MSCs) and circulating skeletal stem/progenitor cells. While BM MSCs are the most investigated and established source for tissue engineering, circulating progenitor cells have garnered attention in regenerative medicine due to their relative ease of isolation and elevated osteogenic potential [4]. Of particular interest are endothelial progenitor cells (EPCs), since a critical step in functional bone healing is the restoration of local blood flow. Recent discoveries have shown an overlap in the progenitor cell lineages giving rise to endothelial and osteoblastic cells [4], as well as the existence of a developmental, osteogenic reciprocity between endothelial cells and osteoblasts [5]. In response to tissue ischemia, EPCs mobilize from the bone marrow into peripheral circulation where they home to bone-healing sites (i.e. fractures or distraction osteogenesis) and promote vasculo-/angiogenesis [6,7]. This is key to the healing process because angiogenic events are one of the limiting factors in bone regeneration and function as the primary regulatory mechanism that directs bony repair [8].

Clinical translation of stem cell therapies for bone regeneration has been shown to be feasible using a variety of techniques [9]. Delivery of MSCs via percutaneous injections or scaffold-based technologies have been demonstrated to have efficacy in mineralization of various osseous defects including fracture nonunion and critical sized cranial defects [10–12]. MSCs have also been used to arrest or reverse the progression of osteonecrosis [13] and achieve high rates of posterior spinal fusion [14].

Although human studies using EPCs have been limited, animal experiments have been successful in regenerating bone using EPCs. Systemic administration of circulating CD34^+^ cells allows for recruitment to the fracture site and enhancement of vasculogenesis and osteogenesis, ultimately leading to clinically functional recovery of skeletal defects [15]. However large systemic doses are likely required for a clinical effect, and these transplanted cells migrate not only to the site of injury but also to the lung, liver, thymus, and brain, potentially causing unforeseen side effects. In an effort to avoid systemic effects, EPCs were subsequently seeded locally into a fracture site. EPC-mediated bone healing was shown to occur in a dose-dependent manner, with higher doses of CD34^+^ cells required for enhanced effects [16]. Local EPC-treated rat femurs had abundant new bone and vessel formation with higher torsional strength and stiffness when compared to controls [17,18]. Similar effects were demonstrated in sheep models where *ex vivo* expanded autologous EPCs were implanted in critical-sized bone defects in sheep [8].

While many of these approaches have demonstrated effective bone regeneration, cell-based therapies require donor tissue sampling, often followed by extensive cell expansion steps before therapeutic implantation. These *ex vivo* cell expansion procedures can be both time-consuming and cost-expensive [3]. Moreover, isolated tissue-derived primary cells are often heterogeneous and difficult to standardize, making the procurement of a reliable, reproducible cell source a major challenge in cell-based approaches [19]. The well-studied BM-MSCs can only be isolated via BM aspiration under anesthesia, which is considered a form of surgical intervention. In contrast, peripheral blood cells are appealing because their aspiration does not require anesthesia and their isolation can be performed in a relatively minimally invasive, safe, and efficacious fashion [4]. However, the time investment alone required in CD34^+^ cell selection and subsequent expansion of EPCs to therapeutic levels, which may require up to weeks, may be a hurdle considered too daunting for some. Concentrated BM may fall into favor in this case, as it can be used at the point-of-care, in a single surgical procedure, without the risks, cost or time expense of *ex vivo* cell expansion [9].

Alternatively, the development of a novel approach of *in situ* tissue generation utilizes the body’s own regenerating capacity by mobilizing host endogenous stem cells or tissue-specific progenitor cells to the site of injury, eliminating any need for *ex vivo* cell manipulation before implantation [20]. Direct targeting of the stem cell niche can induce progenitor cell mobilization in the form of osteoblasts. For example, stimulation of the parathyroid hormone receptor promotes proliferation of osteoblasts and secretion of paracrine factors that, in turn, increases the number of hematopoietic stem cells [19]. However further work is needed to elucidate the appropriate balance in activation of these receptors to avoid hormone overdrive, as well as understanding trafficking control, homing properties, and mechanisms of activation before these methods can be utilized clinically for structural and functional bone regeneration.

Vast strides have been made in making cell-based regenerative technologies a realistic strategy in bone regeneration. Cost and time considerations will ultimately affect the application of stem cell therapies provided that they can demonstrate improved clinical outcomes or decreased hospitalization requirements. Improved methods involving cell selection, effective expansion [21], synthetic mediators to sustain proliferation [22], effective use or reuse of media and growth factors, and 3-dimensional lattices allowing for maximal expansion [23] are needed to develop cost-effective approaches [24].
